# Poly(I:C) preconditioning protects the heart against myocardial ischemia/reperfusion injury through TLR3/PI3K/Akt-dependent pathway

**DOI:** 10.1038/s41392-020-00257-w

**Published:** 2020-11-06

**Authors:** Erya Chen, Chan Chen, Zhendong Niu, Lu Gan, Qiao Wang, Ming Li, XingWei Cai, Rui Gao, Sruthi Katakam, Hai Chen, Shu Zhang, Ronghua Zhou, Xu Cheng, Yanhua Qiu, Hai Yu, Tao Zhu, Jin Liu

**Affiliations:** 1 Department of Anesthesiology and Translational Neuroscience Center, West China Hospital, Sichuan University, Chengdu, Sichuan China; 2 Department of Emergency Medicine, West China Hospital, Sichuan University, Chengdu, Sichuan China; 3 Institute of Cell Engineering, Department of Neurology, School of Medicine, Johns Hopkins University, Baltimore, MD USA

**Keywords:** Innate immunity, Cardiology, Drug development

## Abstract

Emerging evidence suggests that Toll-like receptors (TLRs) ligands pretreatment may play a vital role in the progress of myocardial ischemia/reperfusion (I/R) injury. As the ligand of TLR3, polyinosinic-polycytidylic acid (poly(I:C)), a synthetic double-stranded RNA, whether its preconditioning can exhibit a cardioprotective phenotype remains unknown. Here, we report the protective effect of poly(I:C) pretreatment in acute myocardial I/R injury by activating TLR3/PI3K/Akt signaling pathway. Poly(I:C) pretreatment leads to a significant reduction of infarct size, improvement of cardiac function, and downregulation of inflammatory cytokines and apoptotic molecules compared with controls. Subsequently, our data demonstrate that phosphorylation of TLR3 tyrosine residue and its interaction with PI3K is enhanced, and protein levels of phospho-PI3K and phospho-Akt are both increased after poly(I:C) pretreatment, while knock out of TLR3 suppresses the cardioprotection of poly(I:C) preconditioning through a decreased activation of PI3K/Akt signaling. Moreover, inhibition of p85 PI3K by the administration of LY294002 in vivo and knockdown of Akt by siRNA in vitro significantly abolish poly(I:C) preconditioning-induced cardioprotective effect. In conclusion, our results reveal that poly(I:C) preconditioning exhibits essential protection in myocardial I/R injury via its modulation of TLR3, and the downstream PI3K/Akt signaling, which may provide a potential pharmacologic target for perioperative cardioprotection.

## Introduction

As the most lethal manifestation of coronary heart disease, acute myocardial infarction affects more than 7 million individuals worldwide annually,^1^ accounting for a substantial footprint on global health burden, especially in developing countries. In the initial phase of myocardial infarction, partially or complete occluded coronary deprives the downstream myocardium of nutrients and oxygen, and restoration of blood flow aborts deleterious effect of ischemia. However, reperfusion itself triggers a subsequent wave of insult, termed ischemia/reperfusion (I/R) injury,^2^ which further threatens myocardial recovery and hence aggregates irreversible heart damage in survivors. In response to damage, injured or dying myocardial cells releases damage-associated molecular patterns (DAMPs), which are detected and recognized by resident cardiac immune cells. More recently, it has become common that the innate immune system responds to I/R injury-induced sterile inflammation rapidly and contributes an essential role in modulating the pathological process, though more remains to be elucidated.^3^ Thus, there is considerable interest focus on defining the mechanism of the innate immune system in hopes of mitigating the deleterious effect of reperfusion while maintaining a healthy healing process.

As the first-line defense of the innate immune system, Toll-like receptors (TLRs), one of the pattern recognition receptors, have been reported to recognize DAMPs released during I/R injury. Due to the homeostatic effect by delimiting tissue injuries, TLRs and their ligands appear to be a promoting target with great potential for the treatment of reperfusion injury. In fact, TLRs have participated in the tolerance to ischemic and inflammatory resistance induced by preconditioning. For instance, pretreatment with TLR2 ligand Pam3CSK4 mimicked the cytoprotection of ischemic preconditioning in cardiac I/R injury.^4^ Also, activation of TLR4 by lipopolysaccharide (LPS) preconditioning exhibited reduced infarct size and improved cardiac function in rats, mice, and rabbits.^5^ However, the underlying mechanism of TLRs ligand preconditioning is not entirely understood. For TLR2 and TLR4, some studies have demonstrated that the cytoprotective effect of their ligands preconditioning, may be related with the activation of phosphatidylinositol 3-kinase (PI3K)/Akt signaling pathway, which prevents pro-inflammatory and apoptosis events through cross-talking with nuclear factor (NF)-κB signaling pathway.^6^

The cardioprotection of TLRs-cardiac preconditioning has addressed a cross-talk between TLRs and PI3K/Akt signaling. Within multiple innate immune receptors, TLRs have been identified in a broad series of cell types. Of note, the expression level of messenger RNA for TLR2–4 are nearly tenfold higher than the rest of TLRs in human hearts and cardiomyocytes from neonatal rats.^7,8^ Instead of myeloid differentiation factor 88 (Myd88)-dependent manner in other TLRs, TLR3 engages cytoplasmic adapter TIR domain-containing adapter inducing interferon [IFN]-β (TRIF) and finally induces translocation of NF-κB and activation of interferon-regulatory factor 3 (IRF3), resulted in induction of inflammatory and apoptotic mediators.^9^ Recently, polyinosinic-polycytidylic acid (poly(I:C)), the specific ligand of TLR3, has been demonstrated to be able to reduce cerebral infarct size and increases the survival rate of mice following acute cerebral ischemia damage by activating of TLR3/TRIF pathway, eliciting limitation in the systemic inflammatory response and caspase3 activity.^10–14^ However, the role of poly(I:C) preconditioning on myocardial I/R injury and the related mechanisms remained unexplored.

In the present study, we investigated the effect of poly(I:C) preconditioning on myocardial I/R injury and observed that poly(I:C) preconditioning reduced infarct size and improved cardiac function. Importantly, we demonstrated for the first-time poly(I:C) induced interaction between TLR3 and PI3K in vivo, which was accelerated by treat time and I/R insult. Subsequently, we found that pharmacological inhibition of the PI3K activity in vivo or knockdown of Akt through siRNA transfection in vitro abolished the cytoprotective effect of poly(I:C). Collectively, these findings suggest that poly(I:C) preconditioning enhances the activation of TLR3 and its interaction with PI3K/Akt signaling pathway and thus yields cardiac tolerance against myocardial I/R injury.

## Results

### Poly(I:C) preconditioning ameliorated myocardial I/R injury

**Fig. 1:**
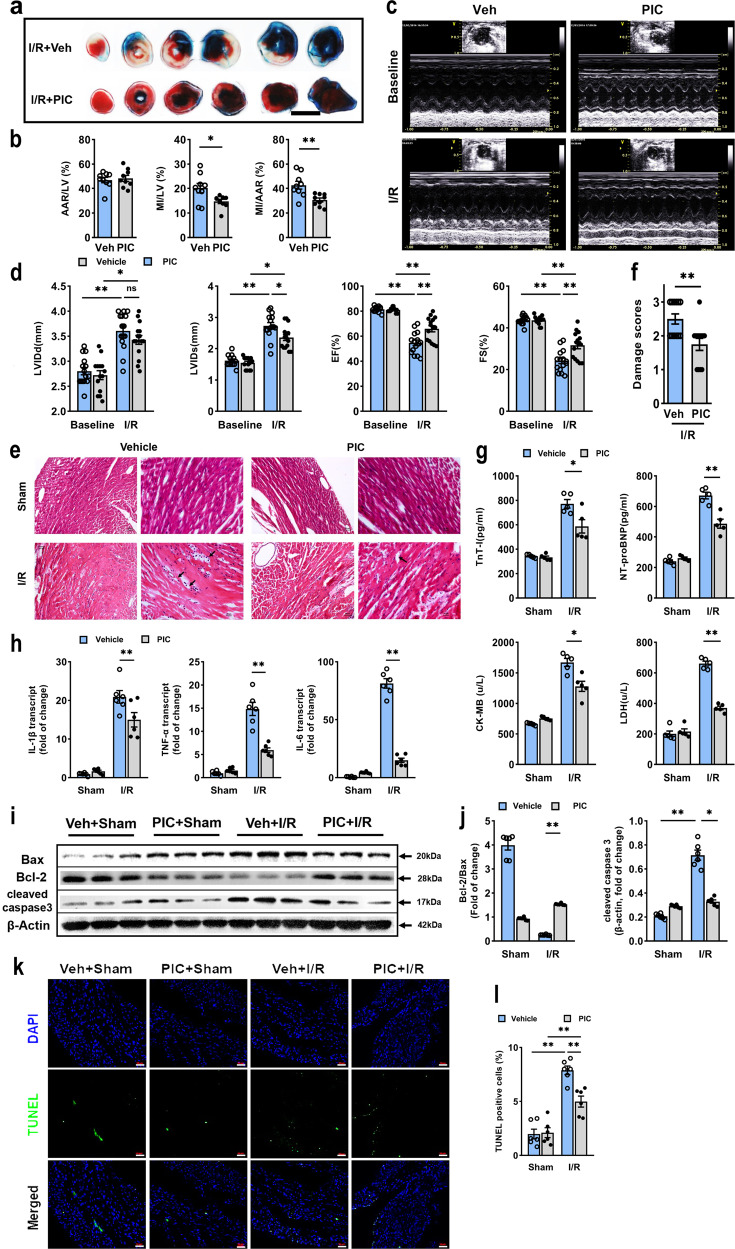
Poly(I:C) pretreatment protected mice hearts from I/R-induced injury via limitation of inflammatory and apoptosis. **a** Representative photographs of TTC-stained, Evans Blue perfused heart sections obtained from poly(I:C) or vehicle pretreated mice subjected to I/R injury (45 min ischemia/24 h reperfusion). Red, AAR; blue, healthy myocardium tissue; white, infarcted tissue; scale bars, 1 mm. **b** Quantitative data of left ventricular infarct size and AAR in poly(I:C) or vehicle pretreated mice (*n* = 9) (experimental groups were compared via unpaired student’s *t* test, bars indicate the SEM, *P < 0.05; **P < 0.01). **c** Representative transthoracic echocardiography of 24 h before and after I/R injury. **d** Average data of LVIDd, LVIDs, EF, and FS measured by echocardiography in poly(I:C) or vehicle pretreated mice subjected to I/R (*n* = 15) (All experiment groups were compared via one-way ANOVA with a Bonferroni’s multiple comparisons test, bars indicate the SEM, *P < 0.05; **P < 0.01). **e** Representative photographs of HE staining heart sections in all groups. Scale bars (first panel of every group): 50 μm; scale bar (second panel of every group): 20 μm. **f** Morphological evaluation of myocardial injury after I/R in each group (*n* = 6) (all experiment groups were compared via unpaired Student’s *t* test, bars indicate the SEM, *P < 0.05; **P < 0.01). **g** The cardiac functional markers in serum were analyzed with an ELISA kit (*n* = 6). **h** Expression of inflammatory cytokine IL-1β, TNF-α, and IL-6 as assessed qRT-PCR in hearts subjected to I/R with poly(I:C) or vehicle pretreatment (*n* = 6). **i**, **j** Representative western blot (**i**) and average data (**j**) for Bax, Bcl-2 and cleaved caspase 3 in mice subjected to sham and I/R with poly(I:C) or vehicle pretreatment (*n* = 6). **k**, **l** Representative microphotographs (**k**) and average data (**l**) for TUNEL assay of heart sections in mice subjected to I/R (*n* = 6). scale bars, 200 μm. (All experimental groups were compared via one-way ANOVA with a Bonferroni’s multiple comparisons test, bars indicate the SEM, *P < 0.05; **P < 0.01). Veh, vehicle; PIC, poly(I:C); LV, left ventricle; I/R, ischemia/reperfusion

Promoted by results of previous researches in which poly(I:C) preconditioning showed a neuroprotective effect against cerebral I/R injury,^11^ we hypothesized that poly(I:C) preconditioning could confer protection of heart subjected to myocardial I/R injury. Since the infarct size corresponds to the risk of developing heart failure, it is inevitable to investigated whether poly(I:C) preconditioning will limit infarct development after I/R injury. Poly(I:C) preconditioning displayed a considerable reduction in the myocardial infarct size ~70% of the vehicle group subjected to I/R, and two groups had commensurate AAR (Fig. 1a, b). Next, we assessed the change of the cardiac function. Echocardiography revealed that the left ventricular end-systolic internal dimension (LVIDs) was slightly decreased in the poly(I:C) preconditioned group compared with the control group. In contrast, no difference in left ventricular end-diastolic internal dimension (LVIDd) was observed between these two groups. Again, poly(I:C) preconditioning exhibited a clear tendency for recovery from cardiac contractile dysfunction, which indicated by increased ejection fraction (EF) and fractional shortening (FS) (Fig. 1c, d). Then, we performed the hematoxylin and eosin (HE) staining to appraise the cardiac pathological changes. Apparently, the poly(I:C) pretreated hearts showed less severe myocardial damage, as evidenced by the relieving edema, steatosis, and subendocardial hemorrhage following myocardial I/R injury (Fig. 1e, f). Furthermore, we analyzed the cardiac biomarkers in serum, which have evolved as essential tools in cardiology and can help to predict adverse cardiovascular events according to recent publications.^15^ In accordance with the above observations, troponin I, N-terminal pro B-type natriuretic peptide (NT-pro BNP), creatine kinase-MB (CK-MB) and lactate dehydrogenase (LDH) were all significantly decreased at 6 h after I/R injury (Fig. 1g). Thus, these data indicated that poly(I:C) treatment before exposure to I/R yielded an infarct-sparing effect.

### Poly(I:C) preconditioning suppressed the inflammatory and apoptotic responses in vivo

As inflammation plays a predictable role in adverse cardiovascular events following I/R injury,^16^ next, we aimed to elucidate whether inflammation response was involved in the effect of poly(I:C) pretreatment. The results of PCR revealed that the transcript level of pro-inflammatory cytokines, such as IL-1β, TNF-α, and IL-6, was markedly reduced in poly(I:C) pretreated mice compared with control ones (Fig. 1h), indicating the inhibition of pro-inflammatory cytokines transcription by poly(I:C) preconditioning. Moreover, apoptosis acts as another vital component of cardiomyocyte death within multiple cardiovascular disease.^17^ Among a wide range of receptors, Bax is generally associated with apoptosis, well Bcl-2 acts as an antiapoptotic regulator.^18^ Therefore, the ratio of Bcl-2/Bax is used to indicate the anti-apoptotic level. We performed western blot and found that the expression of Bcl-2/Bax was enhanced after poly(I:C) pretreatment, and this was correlated with the suppressed expression of apoptosis-related molecule cleaved caspase3 (Fig. 1i, j). To further corroborate this, TUNEL assay was performed, and apoptotic death in cardiomyocytes was notably reduced by poly(I:C) preconditioning (Fig. 1k, l). Taken together, the results suggested that poly(I:C) preconditioning potently inhibited inflammatory response and reperfusion-induced apoptosis.

### Poly(I:C) pretreatment induced cardioprotection through activation of TLR3

**Fig. 2:**
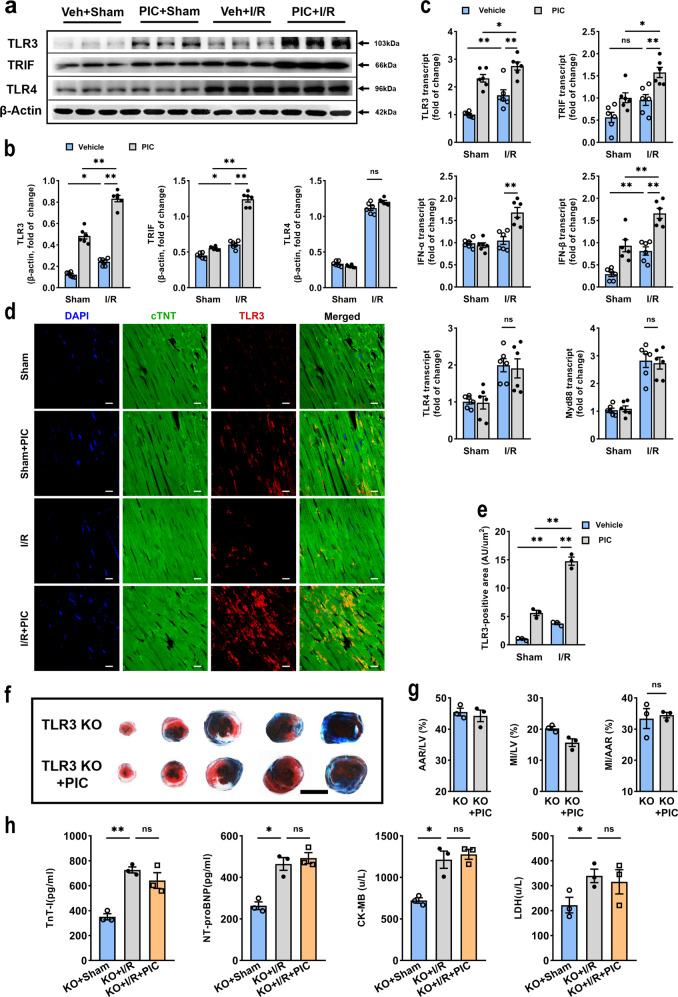
TLR3 activation was responsible for poly(I:C) preconditioning protective effect against I/R injury. **a**, **b** Representative western blot (**a**) and average data (**b**) for TLR3, TRIF, and TLR4 in hearts subjected I/R (*n* = 6). **c** The relative expression of TLR3, TRIF, IFN-α, IFN-β, TLR4, and Myd88, analyzed by qRT-PCR in hearts subjected to I/R with poly(I:C) or vehicle pretreatment (*n* = 6). (All experimental groups were compared via one-way ANOVA, bars indicate the SEM, *P < 0.05; **P < 0.01). **d**, **e** Representative microphotographs (**d**) and average data (**e**) for TLR3 (as assessed by TLR3, cTNT, and DAPI fluoresce intensity) of heart sections in mice subjected to 45 min of myocardial ischemia following 24 h of reperfusion (*n* = 3) (all experiment groups were compared via one-way ANOVA with a Bonferroni’s multiple comparisons test, bars indicate the SEM, *P < 0.05; **P < 0.01). Blue, DAPI; green, cTNT; red, TLR3, scale bars, 50 μm. **f**, **g** Infarct size was identified with TTC-Evans blue double stain in *tlr3*^*−/−*^ mice, scale bars, 1 mm (*n* = 3) (two experimental groups were compared via unpaired student’s *t* test, bars indicate the SEM, *P < 0.05; **P < 0.01). **h** The cardiac functional markers were analyzed with ELISA kit (*n* = 6) (all experiment groups were compared to *tlr3*^*−/*^^−^ mice +I/R group via one-way ANOVA with a Dunnett’s multiple comparisons test, bars indicate the SEM, *P < 0.05; **P < 0.01). TLR3 KO, TLR3 knockout mice; ns not statistically significant

As a synthetic double-stranded RNA, poly(I:C) is recognized and specifically binds to TLR3, then activates downstream transcription factors via its intercellular signaling pathway.^19^ To further confirm the cytoprotective effect of poly(I:C) preconditioning was via TLR3 activation in our current study, we assessed the expression level of TLR3, TLR4, and their downstream adapters. In the presence of I/R stimulus, poly(I:C) significantly upregulated protein expression of TLR3 and TRIF, in contrast, TLR4 expression was not modified at all (Fig. 2a, b). In fact, with regard to mRNA expression of TLR3, TRIF, IFN-α, and IFN-β but not TLR4 and Myd88, were increased in poly(I:C) pretreated mice (Fig. 2c). Consistent with these alterations, immunofluorescent TLR3-positive area was also higher in poly(I:C) pretreated mice than that in controls (Fig. 2d, e). Based on these data, poly(I:C) preconditioning apparently induced TLR3 activation. NF-κB, an innate immune signaling mediator, which is ultimately activated in TLR3 signaling cascade.^20^ As previously reported,^14^ I/R stimuli triggered the binding activity of p65 NF-κB, which significantly decreased with poly(I:C) preconditioned (Supplementary Fig. 2a). Besides, the phosphorylation level of p65 NF-κB was also reduced by poly(I:C) preconditioning in ischemic myocardium tissue (Supplementary Fig. 2b, c). Thus, suggesting that poly(I:C) preconditioning induced TLR3 activation, therefore, responded to I/R injury and modulated tissue damage following I/R insults in hearts.

Since the effect of poly(I:C) preconditioning correlated with activation of TLR3, we further verified this phenomenon in TLR3 knockout mice. No difference was found in infarct size between *tlr3*^−*/*−^ mice pretreated with vehicle and poly(I:C) (Fig. 2f, g), as consistent with the absence of change in cardiac functional markers, such as troponin I, NT-pro BNP, CKMB, and LDH (Fig. 2h), indicating that poly(I:C) directly relieves I/R damage via TLR3.

### Poly(I:C) preconditioning enhanced phosphorylation of PI3K/Akt

**Fig. 3:**
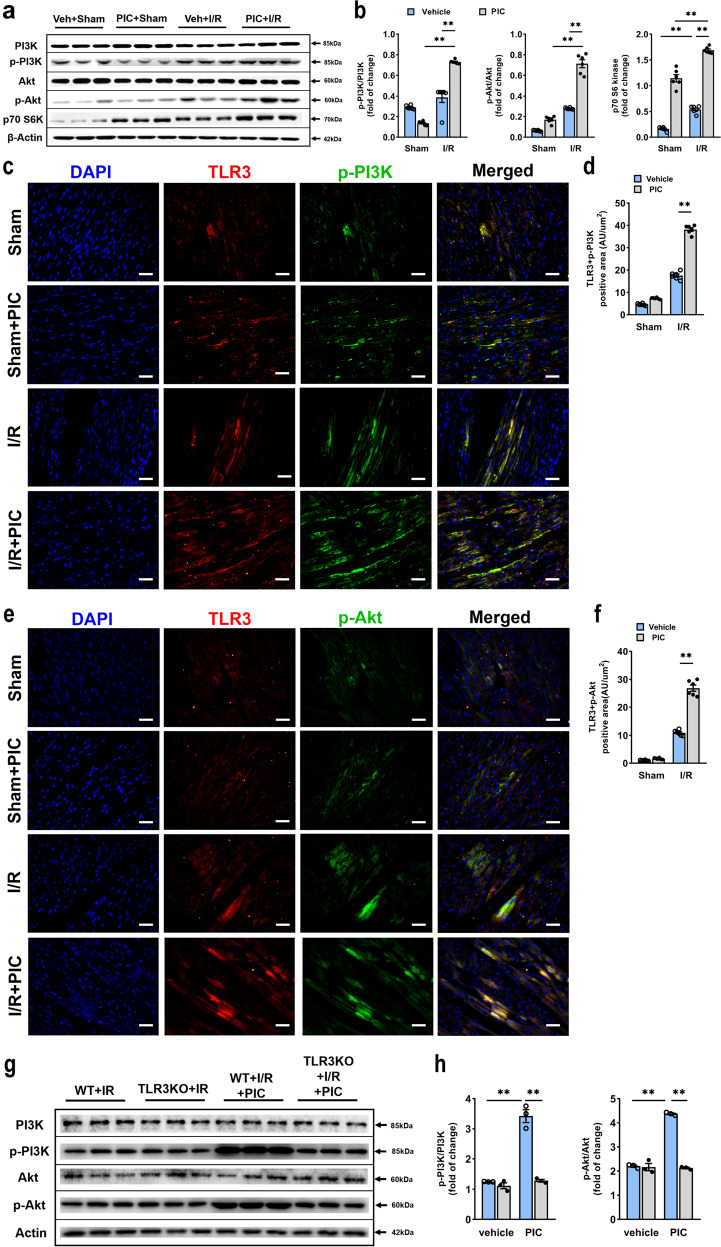
Poly(I:C) pretreatment resulted in PI3K and Akt phosphorylation via TLR3. **a**, **b** Representative western blot (**a**) and average data (**b**) for PI3K, phospho-PI3K, Akt, phospho-Akt and p70 S6 kinase in ischemic myocardium with or without poly(I:C) preconditioning (*n* = 6). **c**, **d** Immunofluorescent colocalization (**c**) and quantification (**d**) of TLR3 and phospho-PI3K in mice subjected I/R with or without poly(I:C) preconditioning, scale bars, 50 μm (*n* = 6). **e**, **f** Immunofluorescent colocalization (**e**) and quantification (**f**) of TLR3 and phospho-Akt in mice subjected I/R with or without poly(I:C) preconditioning, scale bars, 50 μm (*n* = 6) (all experiment groups were compared via one-way ANOVA with a Bonferroni’s multiple comparisons test, bars indicate the SEM, *P < 0.05; **P < 0.01). **g**, **h** Representative western blot (**g**) and average data (**h**) for PI3K, phospho-PI3K, Akt and phospho-Akt in *tlr3*^−*/*−^ mice subjected to I/R with or without poly(I:C) preconditioning (*n* = 6) (all experiment groups were compared with wild-type mice poly(I:C) pretreatment group via one-way ANOVA with a Dunnett’s multiple comparisons test, bars indicate the SEM, *P < 0.05; **P < 0.01). p-PI3K, phosphorylated PI3K; p-Akt, phosphorylated Akt; p70 S6K, p70 S6 kinase

In particular, PI3K and its downstream kinase Akt are activated by TLR ligand through preconditioning mechanism as previously reviewed^6^ and act as negative feedback to limit pro-inflammatory and apoptosis events in response to harmful stimuli. Poly(I:C) preconditioning upregulated phosphorylation of PI3K and Akt, and accordingly, the protein expression level of p70 S6 kinase, a critical downstream target of PI3K/Akt/mTOR pathway, was substantial increased (Fig. 3a, b). Moreover, several studies have identified a cross-talk between TLRs and PI3K/Akt signaling pathway.^21,22^ Therefore, we further explored the colocalization of TLR3 with phospho-PI3K and phospho-Akt. It was apparent that the combination of TLR3 and phospho-PI3K (Fig. 3c, d) or phospho-Akt (Fig. 3e, f) in ischemic heart lesions was more pronounced in poly(I:C) preconditioned mice subjected to myocardial I/R injury. We also detected the phosphorylation levels of PI3K and Akt in *tlr3*^*−/−*^mice, which were significantly decreased compared with poly(I:C) pretreated ones compared with the wile type (Fig. 3g, h). In contrast, there was no TLR3-positive cardiomyocyte observed in *tlr3*^*−/−*^ mice ischemic myocardium tissue (Supplementary Fig. 3). All these findings indicated that poly(I:C) preconditioning promoted activation of PI3K/Akt signaling pathway via TLR3.

### Poly(I:C) pretreatment inhibited inflammatory response and apoptosis cell death in vitro

**Fig. 4:**
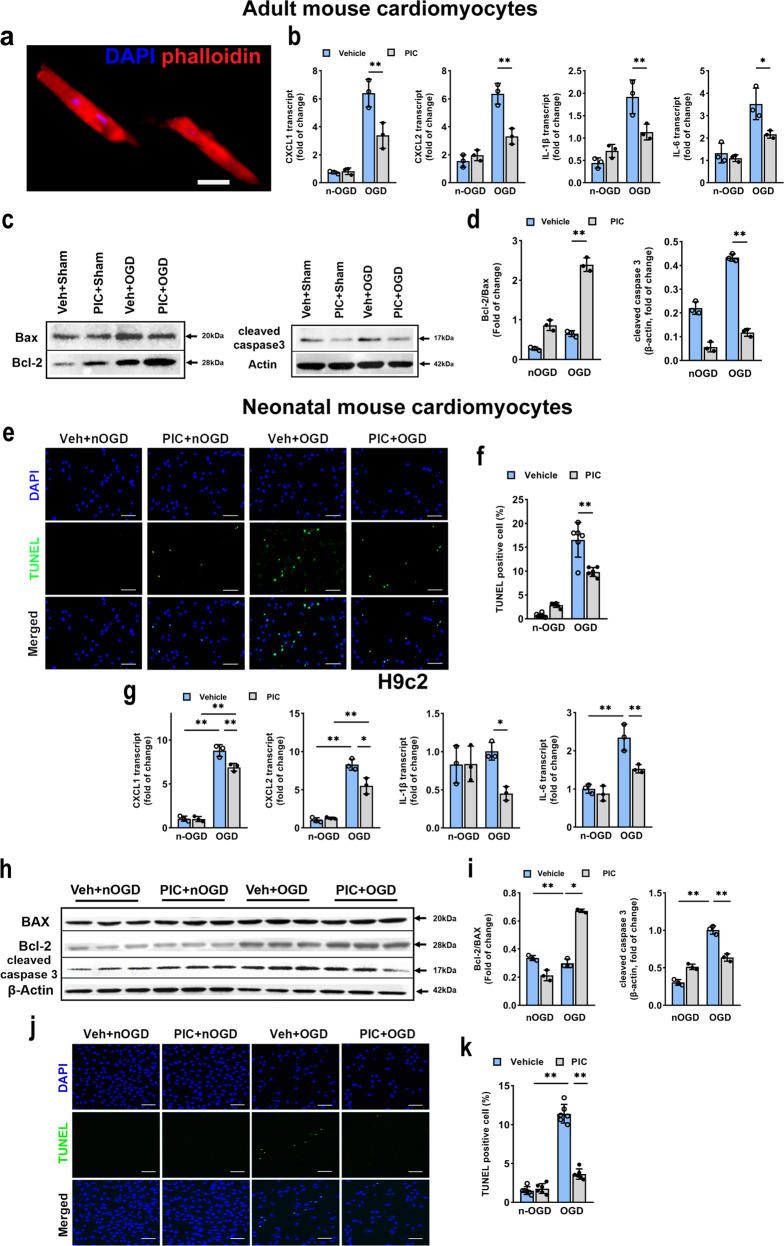
Inflammatory response and apoptosis were inhibited in adult mouse cardiomyocytes and H9c2 cell lines by poly(I:C). **a** Isolated primary cardiomyocytes from adult mice. Blue, DAPI; red, phalloidin-stained microwire skeleton of cardiomyocytes, scale bars, 10 μm. **b** Expression of inflammatory cytokine CXCL1, CXCL2, IL-1β, and IL-6 as assessed by qRT-PCR in adult mouse cardiomyocytes subjected to OGD with poly(I:C) (*n* = 3). **c**, **d** Representative western blot (**c**) and average data (**d**) for Bax, Bcl-2, and cleaved caspase3 in adult mouse cardiomyocytes subjected to OGD with poly(I:C) (*n* = 3). **e**, **f** Representative microphotographs (**e**) and average data (**f**) for TUNEL-stained of primary neonatal mouse cardiomyocytes subjected to OGD with 12 h poly(I:C) preconditioning (*n* = 6). Blue, DAPI; green, TUNEL-positive cardiomyocytes; scale bars, 50 μm. **g** Expression of inflammatory cytokine CXCL1, CXCL2, IL-1β, and IL-6 in H9c2 cell lines subjected to OGD with poly(I:C) (*n* = 3). **h**, **i** Representative western blot (**h**) and average data (**i**) for Bax, Bcl-2 and cleaved caspase3 in H9c2 subjected to OGD with poly(I:C) (*n* = 3). **j**, **k** Representative microphotographs (**j**) and average data (**k**) for TUNEL assay of H9c2 cell lines subjected to OGD with 12 h poly(I:C) preconditioning (*n* = 6) (all experiment groups were compared via one-way ANOVA with a Bonferroni’s multiple comparisons test, bars indicate the SEM, *P < 0.05; **P < 0.01). nOGD, non OGD

The effect of poly(I:C) preconditioning-induced cytoprotection on cardiomyocytes was further studied in vitro by culturing adult mouse cardiomyocyte and H9c2 cell lines. In isolated adult mouse cardiomyocytes (Fig. 4a), poly(I:C) pretreatment significantly attenuated oxygen–glucose deprivation (OGD)-induced the increase in transcript levels of pro-inflammatory cytokines CXCL-1, CXCL-2, IL-1β, and IL-6 (Fig. 4b). Also, poly(I:C) preconditioned adult mouse cardiomyocytes showed a lower level of apoptosis as indicated by increased the ratio of Bcl-2/Bax and decreased expression level of cleaved caspase3 (Fig. 4c, d). Besides, we performed the TUNEL assay in primary neonatal mouse cardiomyocytes (NMCMs) and found that the number of TUENL-positive cells was also notably decreased (Fig. 4e, f). To further investigate whether poly(I:C) preconditioning protects cardiomyocyte, we used an additional cardiac myocyte, H9c2 cell lines, to verify this possibility. The H9c2 cell lines were employed to test different periods of reoxygenation and different poly(I:C) concentration by cell counting kit-8 (CCK8) assay (Supplementary Fig. 4). We chose the 12.5 μg/ml poly(I:C) and 12 h reoxygenation in following H9c2 studies. The results showed that pretreatment with poly(I:C) significantly reduced pro-inflammatory factors transcription level compared with that in controls (Fig. 4g). Next, we also detected the expression of several apoptotic phenotype markers, and we found that poly(I:C) pretreatment suppressed apoptosis by downregulating Bax and cleaved caspase 3 meanwhile upregulating Bcl-2 (Fig. 4h, i). TUNEL assay also provided further supporting data, indicating that the incidence of apoptosis was positively correlated with poly(I:C) preconditioning (Fig. 4j, k). Thus, these data suggested that poly(I:C) preconditioning might limit cell death by inhibiting OGD-induced inflammatory and apoptosis responses, as consistent with the results in vivo.

### Pharmacological inhibition of PI3K or Akt depletion abolished the protective effect of poly(I:C) preconditioning

**Fig. 5:**
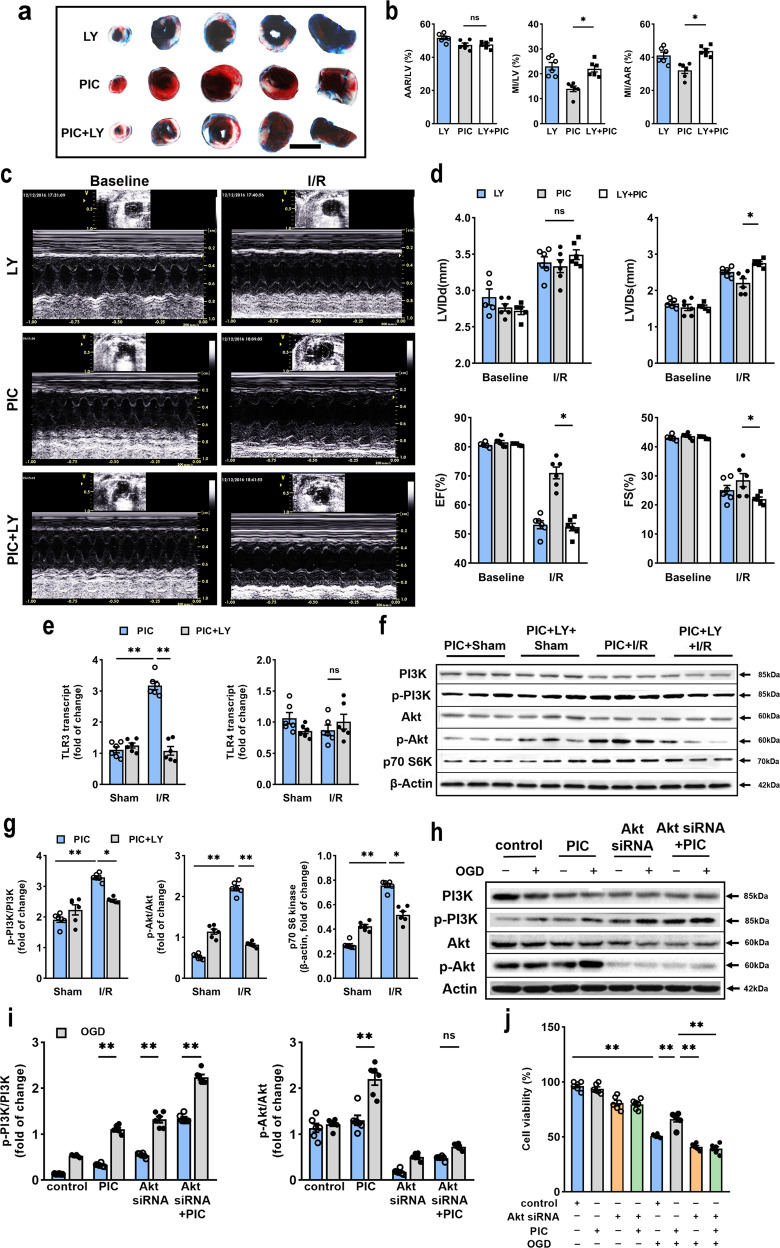
Inhibition of PI3K or transfection of Akt siRNA abolished poly(I:C)-induced cardioprotection following myocardial I/R injury. **a** Representative photographs of TTC-stained, perfused heart sections obtained from poly(I:C) with or without LY294002 pretreated mice subjected to I/R injury, scale bars, 1 mm. **b** Quantitative data of left ventricular infarct size (MI) and area at risk (AAR) in poly(I:C) with or without LY294002 pretreated mice (*n* = 6). **c** Representative transthoracic echocardiography of 24 h before and after I/R injury with or without LY294002 pretreated. **d** Average of data LVIDd, LVIDs, EF, and FS measured by echocardiography in poly(I:C) or vehicle pretreated mice subjected to I/R (*n* = 6) (all experiment groups were compared via one-way ANOVA with a Bonferroni’s multiple comparisons test, bars indicate the SEM, *P < 0.05). **e** Expression of TLR3 and TLR4 as assessed by qRT-PCR in hearts subjected I/R after poly(I:C) preconditioning with or without LY294002 pretreatment (*n* = 6). **f**, **g** Representative western blot (**f**) and average data (**g**) for PI3K, phospho-PI3K, Akt, phospho-Akt, and p70 S6 kinase in hearts subjected to I/R with poly(I:C) and/or LY294002 pretreatment (*n* = 6) (all experiment groups were compared with poly(I:C) I/R group via one-way ANOVA with a Dunnett’s multiple comparisons test, *P < 0.05; **P < 0.01). **h**, **i** Representative western blot (**h**) and average data (**i**) for protein level of phospho-PI3K, PI3K, phospho-Akt, and Akt in neonatal cardiomyocytes transfected with Akt siRNA and poly(I:C) before OGD (*n* = 6). **j** Cell viability was detected in neonatal cardiomyocytes transfected with Akt siRNA for 24 h and received OGD performance (*n* = 6) (all experimental groups were compared via one-way ANOVA with a Turkey’s multiple comparisons test, bars indicate the SEM, *P < 0.05; **P < 0.01). LY, LY294002

As shown in Fig. 3, the western blot and immunofluorescent staining revealed the occurrence of poly(I:C) preconditioning-induced cardioprotection was correlated with the PI3K/Akt pathway. To determine the role of the PI3K/Akt pathway in the cytoprotective effect, the specific PI3K inhibitor, LY294002, was utilized. LY294002 was injected i.p. 15 min before myocardial ischemia. As it was shown in Fig. 5a, b, LY294002 completely abolished reduction in myocardial infarct size induced by poly(I:C) preconditioning. Also, LY294002 reversed the effect in cardiac function caused by poly(I:C) preconditioning (Fig. 5c, d). Moreover, no statistically significant changes were observed in the transcript level of pro-inflammatory cytokines (Supplementary Fig. 5a). Moreover, LY294002 suppressed the activation of TLR3 and downstream receptors, except IFN-α, while the expression of TLR4 and Myd88 did not show any significant change (Fig. 5e, Supplementary Fig. 5b). Moreover, inhibition of PI3K led to a reduction in the phosphorylation level of PI3K and Akt, which were promoted by poly(I:C) pretreatment, along with the protein level of p70 S6 kinase (Fig. 5f, g). These results implied that the PI3K/Akt signaling pathway might mediate the protective effect of poly(I:C) preconditioning, while its inhibitor, LY294002, could block this effect.

According to the reference, Akt, the downstream kinase of PI3K, can transduce enzymes protecting the myocardium from I/R injury.^23^ As Akt1 is ubiquitously expressed and its critical role in cell survival,^24,25^ we further generated Akt1 knockdown cardiomyocytes through the transfection of Akt1 siRNA. We have found the expression of phospho-PI3K in neonatal mice cardiomyocytes significantly increased, while level of Akt were decreased and phospho-Akt were hardly detected treated with or without poly(I:C) (Fig. 5h, i). Also, we examined the ratio of dead cells and found that Akt depletion exacerbated cell death after OGD insult and was unable to rescue by poly(I:C) pretreatment (Fig. 5j). Thus, we believed that Akt is required for the cytoprotective effect of poly(I:C) preconditioning against OGD damage.

### Poly(I:C) activated TLR3 and induced its enhanced interaction with PI3K

**Fig. 6:**
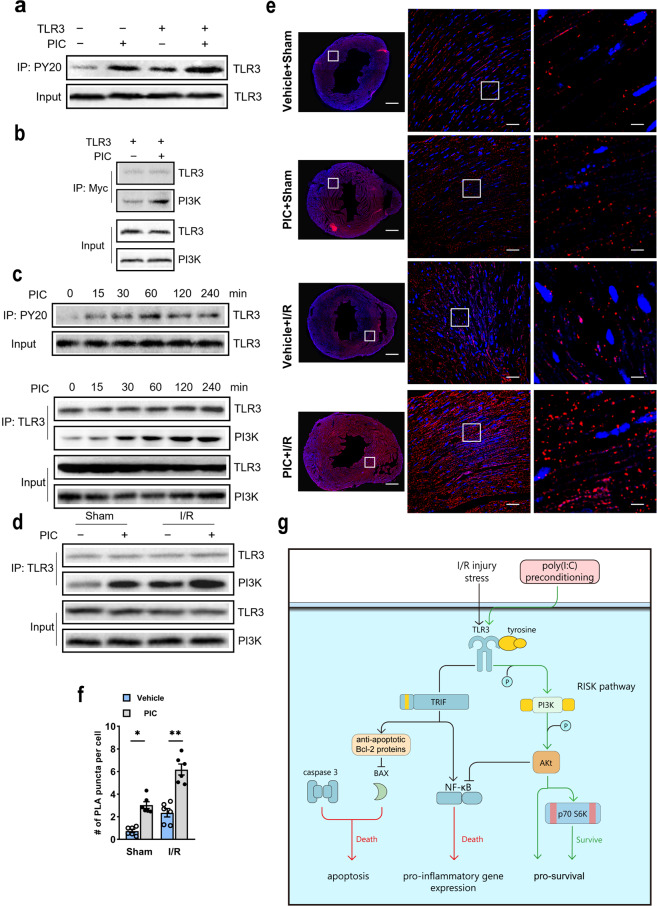
Poly (I:C) increased phosphorylation of TLR3 tyrosine residue and following recruitment of PI3K. **a** HEK 293 cells expressing Myc-tagged TLR3 and treated with poly(I:C). Cell lysates were immunoprecipitated with anti-phosphotyrosine (PY20) and western blotted with anti-TLR3. **b** Cell lysates were immunoprecipitated with anti-Myc and western blotted with anti-PI3K-p85-subunit. The same blot was reprobed with the TLR3 antibody as an immunoprecipitation control. **c** Mice were injected with poly(I:C), and heart tissues were harvested at 0, 15, 30, 120, and 240 min. Samples were immunoprecipitated with antiphosphotyrosine (PY20) and western blotted with anti-TLR3 (upper). Furthermore, samples were immunoprecipitated with anti-TLR3 and western blotted with anti-PI3K. The same blot was reprobed with the TLR3 antibody as immunoprecipitation control (below). **d** Mice were pretreated with or without poly(I:C) and subjected to I/R. Twenty-four hour after I/R heart tissues were taken, and samples were immunoprecipitated with anti-TLR3 and western blotted with anti-PI3K. The same blot was reprobed with TLR3 antibody as an immunoprecipitation control. **e**, **f** Representative photograph (**e**) and quantification (**f**) of PLA reaction between TLR3 and PI3K were shown (*n* = 6) (all experiment groups were compared via one-way ANOVA with a Turkey’s multiple comparisons test, bars indicate the SEM, *P < 0.05; **P < 0.01). Scale bar (first line), 100 μm; scale bar (second line), 50 μm; scale bar (third line), 10 μm. **g** The schematic figure revealed poly(I:C) protects the hearts against I/R injury through phosphorylation of TLR3 tyrosine residue, activation of PI3K and recruitment of Akt, resulting in decreased of NF-κB activity, and reduced inflammatory and apoptotic responses

Considering the close link between TLR3 and PI3K/Akt signaling pathway according to the results mentioned above, a separate set of immunoprecipitation experiments was designed to determine whether TLR3 interacts with PI3K in vitro and in vivo. TLR3 overexpression resulted in a significant increase of tyrosine residue phosphorylation after poly(I:C) stimulation in HEK 293 cells compared with corresponding controls (Fig. 6a). Next, we found that the TLR3-PI3K interaction exhibited a poly(I:C)-dependent fashion and was similar to the kinetics of TLR3 tyrosine residue phosphorylation (Fig. 6b). Besides, we observed that poly(I:C) treatment stimulated TLR3 tyrosine residue phosphorylation, resulting in increased association with PI3K when mice were injected with poly(I:C) for 0, 15, 30, 60, 120, and 240 min (Fig. 6c). More importantly, we detected the interaction between TLR3 and PI3K in mice subjected to I/R injury, and the poly(I:C) preconditioning markedly increased the TLR3–PI3K association compared with that in controls (Fig. 6d). To acquire a better location of TLR3 and PI3K interactions, we utilized proximal ligation assay (PLA), which directly identified protein interactions by fluorescence. Here, we demonstrated a positive PLA reaction between TLR3 and PI3K, and the number of reactions was much higher in the poly(I:C) preconditioning group (Fig. 6e, f), consistent with the immunoprecipitation results. In summary, our data revealed that TLR3 was activated by triggering the phosphorylation of tyrosine residue and followed by increased interaction with PI3K after poly(I:C) pretreatment.

## Discussion

Our findings showed that poly(I:C) pretreatment group had significantly reduced myocardial infarction and better-preserved cardiac function after myocardial I/R injury compared with the control group. Also, we have found that poly(I:C) preconditioning could significantly decrease the expression levels of pro-inflammatory cytokines and apoptosis-related molecules after myocardial I/R injury. However, the cardioprotection of poly (I:C) was lost in TLR3 knockout mice, suggesting that TLR3 is essential for poly (I:C)-induced protective effects. Unlike its critical role in viral myocarditis and distinctly different from the mechanisms of cardioprotection observed in TLR3 knockout mice, poly(I:C) preconditioning exhibits myocardial protection via phosphorylation of TLR3 and its enhanced interaction with PI3K and downstream molecules. Moreover, inhibition of p85 PI3K by the administration of LY294002 in vivo and knockdown of Akt by siRNA in vitro could significantly abolish poly(I:C)-induced cardioprotection. Therefore, we concluded that poly(I:C) preconditioning could protect the myocardium against I/R injury in a TLR3/PI3K/Akt-dependent way (Fig. 6g).

Recently, increasing evidence indicates that TLRs family and their ligands could be the high potential therapeutic targets in cardiac I/R injury. Among all the TLRs ligands, poly(I:C) has exhibited neuroprotective effects on cerebral I/R injury, and its protection is mainly mediated by a TLR3-mediated mechanism,^10,12,14^ specifically, by inhibition of Fas/FADD interaction, activation of TRIF pathway, and downregulation of TLR4 signaling.^11,13^ However, to the best of our knowledge, the role of poly(I:C) preconditioning in myocardial I/R injury and the related mechanisms have not been reported previously. With an increased aging population worldwide, there could be more aged patients at high risk of ischemic cardiac damage due to coronary diseases and surgical procedures. Our study regarding the cardioprotection of poly(I:C) as an antecedent treatment may help provide potential benefits to patients who undergo major surgery. Indeed, as a synthetic dsRNA, poly(I:C) has been applied as a clinical therapy for humoral immune-related disease and developed into a promising cancer vaccine adjuvant,^26^ which gives the protective effect of poly(I:C) a promising translation prospect.

In the current study, activation of TLR3 and its downstream signaling through poly(I:C) preconditioning was shown to have similar cardioprotection as that has been observed in the TLR3 deficient mice.^27^ This phenomenon has also been reported in other TLRs. For instance, *tlr4*^*−/−*^ mice and exhibited decreased infarct size compared with wile type after myocardial I/R injury.^28^ While studies also found that a small dose of TLR4 ligand, LPS or LTA, preconditioning yields comparable cardioprotection.^29,30^ Likewise, this also applies to TLR2.^31,32^ Also, back to 1986, serial occlusions of left descending coronary artery, namely ischemic preconditioning, have been found to exert an enormously powerful anti-infarct effect, reducing infarct size to 75%.^33^ The underlying principle of both ischemic and TLRs ligands preconditioning may be related to the adaptive responses to sublethal ischemic/innate immune stimulus and confers a tolerance state so that the myocardium is rendered resistant against a subsequent, more severe ischemic insult.

Of note, different cardioprotective mechanisms have been observed in TLR-deficiency mice and TLRs ligand preconditioning. For example, the cardioprotective mechanism underlying *tlr4*^*−/−*^ mice is mainly related to blunting I/R-induced NF-κB binding activity, which can significantly improve the recovery of cardiac function and downregulated inflammatory cytokine gene expression, suggesting that the TLR4-mediated NF-kB signaling pathway contributed to I/R injury.^34^ However, the cardioprotection induced by TLR4 ligand preconditioning was mediated through activation of the PI3K/Akt signaling pathway.^35^ Besides, mice with TLR2 deficiency exhibited smaller infarct size and more preserved cardiac function by limiting leukocyte influx, cytokine production, and proapoptotic pathway.^36^ Nevertheless, the study about cardioprotection of TLR2 ligand preconditioning found a different mechanism from that of the TLR2 knockout research.^24^ In our study, unlike the mechanism found in TLR3 deficiency-induced cardioprotection, which mainly through inhibition of the TRIF downstream signalings, poly(I:C) preconditioning exhibited cardioprotective effects have been observed mainly through activation of TLR3 and its enhanced interaction with PI3K and downstream signaling.

As an essential pro-survival pathway, PI3K/Akt protein cascades signaling pathway plays an essential role in the development of cardioprotection against myocardial infarction and I/R injury. Importantly, we have found increased TLR3 tyrosine residue phosphorylation and interaction with PI3K in both HEK293 cells and heart subjected to OGD or I/R injury after poly(I:C) stimulation, and the PLA data also clearly showed the enhanced interaction between TLR3 and PI3K induced by poly(I:C) preconditioning in I/R injured myocardium. Besides, overexpression of Akt reduces infarct size in the rat heart,^37^ and transfection of Akt siRNA exacerbated OGD-induced NMCMs death and eliminated protective of poly(I:C), suggesting Akt as an essential pro-survival protein in cardioprotection. The activation of the PI3K/Akt signaling pathway can limit the pro-inflammatory response and apoptosis,^38^ which also was verified by the reduction in the transcript level of pro-inflammatory cytokines and protein expression level of apoptosis-related molecules in our study. Furthermore, Akt phosphorylates downstream protein, such as mTOR/p70 S6 kinase (S6K), has been linked protective effect of ischemic preconditioning.^39^ Kim et al.^40^ found that overexpression of S6K markedly reduced the activity of NF-κB, upon stimulation of TLR ligand. Similarly, in our study, we have found a higher level of p70 S6K and decreased binding activity of p65 NF-κB in the poly(I:C) preconditioning I/R group compared with the control group. Therefore, we speculated that pretreatment of poly(I:C) increased phosphorylation of TLR3 with subsequent recruitment of the p85 subunit of PI3K, and activation of the phosphorylation of Akt-dependent signaling pathway, which led to increased p70 S6K production, reduced inflammatory and apoptotic response, and inhibited p65 NF-κB activity, inducing a cardioprotective phenotype.

Interestingly, for the mRNA and protein expression levels of TLR4 and downstream adapter Myd88, we did not find any difference between the poly(I:C) and vehicle pretreated group subjected to I/R injury. However, this is not consistent with that has been found in cerebral I/R injury, in which poly(I:C) pretreatment has been reported to exhibit cardioprotection by downregulating TLR4 signaling via TLR3. For the protection induced by poly(I:C) preconditioning, unique mechanisms that have existed in different organs remain elusive. In our study, we found that the mRNA expression levels of TRIF and IFN-β were both increased in poly(I:C) I/R group compared with the vehicle I/R group, indicating activation of TLR3/TRIF/IFN signaling. Also, LY294002 inhibited the transcript level of TRIF, IFN-α, and IFN-β after myocardial I/R injury. Therefore, our data indicated that the poly(I:C) preconditioning-induced cardioprotective effect was primarily due to activation of the TLR3/TRIF pathway and its cross-talk with the PI3K/Akt pathway.

Admittedly, our findings must be regarded within the restrictions of various other potential limitations. Firstly, ideal cardioprotection drugs must have short- and long-term effects. However, we only examined the infarct volume and left ventricular function 24 h after I/R injury, representing only the acute post-infarct period. A full study of the final infarcts ends and remodeling (which happens on a much longer time scale) will require further research to study how long the window of protection induced by poly(I:C) preconditioning lasts. Second, to promote the translation of drugs from the bench to clinical trials, at least two animal models are needed to test the effect of drugs. Our group is now working on a rat myocardial I/R model with poly(I:C) preconditioning, completing the validity of poly(I:C) preconditioning in several signaling pathways mediating tolerance to I/R injury. Third, we did not compare the protective effects of poly(I:C) with other TLRs ligands preconditioning in the mouse model of myocardial I/R injury. Still, further study is needed to figure out the common key mediator for the TLR ligands preconditioning.

## Conclusions

In summary, our data indicate that TLR3 ligand preconditioning induced cardiac protection, which is mediated through activation of PI3K/Akt signaling pathway, to reduce overexpression of inflammatory and apoptotic response. These findings further support the hypothesis that TLR3 is essential for mediation of the tolerance against myocardial I/R injury. They also support the hypothesis that the response observed in poly(I:C) preconditioning wherein the poly(I:C) exposure leads to a reprogrammed TLR3 signaling pathway in response to I/R injury to produce protection. Understanding the association between poly(I:C) preconditioning and downstream signaling molecules would highlight the role of TLR3 during myocardial I/R injury and contribute fundamental scientific perception for therapeutic methods. Our data will hopefully support the application of poly(I:C) as a potential preventive and therapeutic target for perioperative cardioprotection.

## Materials and methods

### Experimental animals

The *tlr3*^−/−^ mice were purchased from the Jackson Laboratory and backcrossed to C57BL/6 mice for more generations. Male C57BL/6 mice (8–12 week old, 20–23 g) were purchased from Dossy Animals, China. Animal procedures were conducted according to the Center for Experimental Animals of West China Hospital of Sichuan University Guidelines and were approved by the Animal Care Committee of West China Hospital of Sichuan University (Permit No. 2015025A), and conformed to the Guide for the Care and Use of Laboratory Animals published by the US National Institutes of Health (NIH Publication No. 85-23, revised 1996).

### Experimental design

We employed a preconditioning regimen to investigate the effect of poly(I:C) on myocardial I/R (Supplementary Fig. 1a). C57BL/6 mice were divided into four groups: the vehicle sham group (*n* = 20 per group), the poly(I:C) sham group (*n* = 20 per group), the vehicle I/R group (*n* = 30 per group) and the poly(I:C) I/R group (*n* = 30 per group). The poly(I:C) (Enzo, 0205160) was dissolved in 0.9% sterile saline to reach a concentration of 1 μg μl^−1^. We found the 12.5 mg kg^−1^ dose of poly(I:C) produced the best effect among different doses (5, 12.5, and 25 mg kg^−1^, data not shown). Poly(I:C) were i.p. injected 12 h before ischemia (45 min) followed by 24 h reperfusion without washing out.

The PI3K inhibitor LY294002 (Calbiochem, 440202) was applied to investigate the underlying mechanism related to the TLR3/PI3K signaling pathway. Subjects separated into the poly(I:C) sham group, the poly(I:C) and LY294002 sham group, the poly(I:C) I/R group, and the poly(I:C) and LY I/R group (*n* = 20/group). The LY294002 groups accepted injection i.p. 0.03 mg g^−1^ 15 min before ischemia (45 min) without washing out. Every pretreatment was blind to the researchers during all experiments and data analysis.

### Cell culture and OGD

The H9c2 (2-1) cells were cultured in Dulbecco’s modified Eagle’s medium(DEMEM; Gibco,12430104) containing 4 mM l-glutamine, 4.5 g l^−1^ glucose, penicillin/streptomycin (100 U/ml) and 10% fetal bovine serum (Thermo Fisher, A2720803), and incubated at 37 °C in a humidified chamber with 5% CO_2_ and 95% O_2_. The cells were not allowed to grow to 100% confluency to avoid the loss of differentiation potential. To establish the myocardial I/R injury model in vitro, OGD was performed as previously described.^29,41^ Cells were cultured in glucose-free and serum-free DMEM (Gibco,11966025) in an anaerobic environment with 1% O_2_, 5% CO_2_, and 94% N_2_ at 37 °C for 4 h. The cells were then replaced the glucose-free DMEM with standard culture media and subjected to normoxic conditions for reoxygenation for 3–12 h (Supplementary Fig. 1b). The H9c2 cells only applied in the experiment of the OGD period and poly(I:C) pretreatment concentration. 12 h before OGD, the cells were pretreated with different concentrations of poly(I:C) (0.1–20 μg ml^−1^) or vehicle without washing out. Cell viability was determined by Cell Counting Kit-8 (MedChemExpress, HY-K0301) following the manufacturer.

HEK293 cells were incubated at 37 °C in mediated DEME containing 10% fetal bovine serum in 5% CO_2_ incubator. Plasmid pECMV-TLR3-m-FLAG (Hanbio, generated) was diluted in serum-free DMEM and mixed with polyethylenimine (PEI; Polysciences, 23966-2), administered to cells for 48 h. After that, the media was changed into complete culture media with poly(I:C) (100 μg ml^−1^), 1 h after poly(I:C) pretreatment, the cells were challenged with OGD performance according to previously mentioned.^42^

Mouse adult cardiomyocytes were isolated as previously described.^43^ After anesthesia, the heart was cut and immediately injected with EDTA buffer into right ventricular meanwhile descending aorta was clamped. Then heart was transferred to fresh EDTA buffer in a 60-mm dish and digested with prepared perfusion and digestion buffer by injection into left ventricular. The heart tissue was separated and pulled into 1-mm pieces, gently triturated to dissociated cells. The digestion was stopped by the addition of stop buffer and cell suspension was filtered. The calcium level was gradually restored by four rounds of gravity setting by using calcium reintroduction buffers. Then the cell pellet was resuspended with pre-warmed culturing medium and plated onto precoated culture plastic in a humidified culture incubator with 5% CO_2_ at 37 °C.

The OGD time period of mouse adult cardiomyocytes were 1 h and reoxygenation for 2 h.

Neonatal mouse cardiomyocytes (NMCMs) were isolated as described.^44^ Hearts from 1 to 3 days old C57BL/6 mice were harvested and sheared into small pieces. Heart tissues were incubated in 5 ml Eppendorf tubes containing 2 ml digestion buffer (0.3 mg ml^−1^ collagenase II and 0.45 mg ml^−1^ pancreatin) 30 min at 37 °C. Heart tissues roughly triturated and isolated cardiomyocytes were collected in tube containing medium to inhibit the enzyme activity. The procedures were repeated until heats were completely digested. Then isolated cells in the medium were filtered in 100 μm cell strainer, centrifuged 1.5 min at 1200 rpm and the pellet was resuspended in 2 ml medium. Isolated cells were cultured in culture medium containing 4 mM l-glutamine, 4.5 g l^−1^ glucose, penicillin/streptomycin (100 U ml^−1^) and 10% fetal bovine serum and incubated at 37 °C in a humidified chamber with 5% CO_2_.

### Myocardial I/R

The myocardial I/R procedure was performed with occlusion of the left anterior descending coronary artery (LAD) as previously employed.^24^ Generally, mice were anesthetized with ketamine [120 mg kg^−1^, intraperitoneally (i.p.)] and xylazine (4 mg kg^−1^, i.p.). The depth of anesthesia was evaluated by corneal and withdrawal reflexes. Subjects were intubated and ventilated with 80% oxygen mixed with 20% carbon dioxide employing a rodent ventilator at a rate of 100–120 breaths/min and a tidal volume between 150 and 200 μl. Then, an incision in the skin and a left anterior thoracotomy through the 3,4 intercostal regions was performed. The LAD was carefully exposed and occluded with a 6-0 polypropylene suture and a small tube 2–3 mm from the tip of the left auricle. The tube was gently removed, and the suture was untied (onset of reperfusion) after 45-min period ischemia. The skin was closed, and the intratracheal tube was simultaneously removed. Sham mice underwent a thoracotomy, but the LAD was not occluded. Electrocardiogram was used to monitor heart rate, ensure the LAD was ligated successfully, as well as show any reperfusion-related wave changes within the process of reperfusion (Supplementary Fig. 1c). Rectal temperature was monitored to maintain the body temperature between 36.5 and 37.5 °C during the procedure.

### ELISA

The blood from I/R mice was obtained by heparin-coated syringe and centrifuged 2000 rpm/min for 15 min. Supernatants were collected to quantify the cardiac functional markers troponin I (Tn-I) and N-terminal pro-brain natriuretic peptide (NT-proBNP) with mouse Tn-I ELISA kit (Bioswamp, MU30421) and mouse NT-proBNP ELISA kit (Bioswamp, MU30252) according to the protocol.

### Estimation of myocardial infarct size

After 24 h reperfusion, animals were anesthetized as previously described and sacrificed with the LAD re-occluded, the blood was collected with a 1 ml syringe, then Evans blue (Sigma, E2129) was injected quickly into the left ventricular cavity. During the beating, the dye circulated and distributed equally through the whole heart. Then the left ventricle was harvested and rinsed with ice-cold phosphate buffer saline (PBS), frozen at −20 °C for 30 min, cut into 2 mm-thick transverse slices, then incubated at 37 °C for 20 min in 2% TTC (2,3,5-triphenyl tetrazolium chloride; Sigma, 17779) and 24 h in 4% paraformaldehyde (Biosharp, BL539A). The heart slices were photographed. Digital images were quantified, and risk and infarct sizes in two slides of each slice in cubic centimeter were calculated. The left ventricular size, area at risk size (AAR), and myocardial infarct sizes (MI) of each slice were summed to obtain to evaluate the whole heart infarct sizes. Infarct size was calculated as the percent of MI/AAR for any hearts.

### Echocardiographic assessment of left ventricular structure and function

Animals were anesthetized as previously described before the examination. Echocardiography (Vivid7 Dimension, GE) was used to evaluate mice left ventricular geometry and function with two-dimension guided M-mode. Various parameters were measured, including heart rate, LVIDd, and LVIDs. FS was determined as ([LVIDd − LVIDs]/LVIDd) × 100%. EF was defined by the formula ([end-diastolic volume (EDV) − end-systolic volume (ESV)]/EDV) × 100%. EDV and ESV were measured with the area–length method in a two-dimensional parasternal long-axis view.

### TUNEL staining

The TUNEL immunofluorescence staining was applied to evaluate and analyze the level of apoptosis in the I/R cardiomyocytes. The animals were anesthetized and perfused with cold PBS and 4% paraformaldehyde (*n* = 6/group). The hearts were then sectioned into 20 mm-thick frozen slices. The staining was performed using an DNA Fragmentation Detection Kit (Millipore, QIA39-1EA) according to the protocols. The paraffin-embedded heart sections were first deparaffinized, permeabilized with 20 μg/ml proteinase K in 10 mM Tris-HCl for 15 min and blocked with 10% normal goat serum in PBS at room temperature for 1 h. The stained sections were photographed with a Fluo view FV10i confocal microscope. The nuclei were stained blue with DAPI, and the apoptotic cells were green for TUNEL positive. We counted the number of DAPI and TUNEL-positive cells. Each section was first separated into four quadrants, and the number of positive cells in each quadrant were counted, then averaged. The percentage of TUNEL positive cells was calculated with the formula: green/blue*100%.

### Histology

After collecting blood and rinsing with cold PBS, the hearts (*n* = 6/group) were sliced into five or six equal sections (5 mm). The specimens were fixed in 4% paraformaldehyde, enclosed in paraffin, stained with hematoxylin and eosin (Baso, BA-4025) according to the standard protocols, and examined by light microscopy for histology changes. A scoring system was used to evaluate the histological myocardial damage based on the modified scoring system described by Hu et al.^45^ Simply, myocardial lesions contains interstitial edema, myofiber degeneration (including myofiber swelling and myofibrillar lysis), and subendocardial hemorrhage, as which were all graded according to their severity (0 = no lesion, 1 = mild, 2 = moderate, 3 = marked) and distribution (0 = no damage, 1 = focal damage, 2 = multifocal damage, 3 = diffuse damage). A mean score for each variable was determined for each heart, and a group means the score was calculated.

### Immunofluorescence staining

Hearts were harvested, fixed, and sectioned into 5 mm-thick frozen slices (*n* = 3/group). Then the slides were then blocked with albumin bovine V (Gentihold,10735094001), washed again, and incubated with the anti-TLR3 antibody used at 1:20 dilution 1% BSA for 2 h at room temperature. Next, the slides were washed and incubated in the dark for 45 min at room temperature with fluorescein-conjugated secondary antibodies (Invitrogen, A-11008) used at 1:500 dilution. Nuclear staining was done with DAPI (Beyotime, C1005) and myocardial fiber was stained with cardiac troponin T (Proteintech,11513-1-AP) for 45 min in the dark. The slides were washed, coverslips were mounted, then examined under a fluorescence microscope. Images were taken using Zeiss LSM 710 confocal microscope by simultaneous recording in the 488, 555, and 560 channels as appropriate.

### Quantitative real-time PCR

Ischemic myocardial tissue was harvested quickly 24 h after reperfusion (*n* = 6/group). Total RNA was extracted from the cardiac tissue or cell cytokine using Trizol (Invitrogen, 15596026) according to the instruction. RNA was reverse-transcribed from 2 μg of total RNA by 200U of M-MuLV reverse transcriptase. Real-time PCR was carried out on a Bio-Rad iCycler in 96-well plates and performed with diluted cDNA using primers for tumor necrosis factor-α (TNF-α), interleukin-1β (IL-1β), interleukin-6 (IL-6), TLR3, the adapter protein TIR domain-containing adapter-inducing interferon (TRIF), interferon-beta (IFN-β), interferon-α (IFN-α), toll-like receptor 4 (TLR4), Myd88 (sense and antisense, see in Supplementary Table 1). The products were resolved on 1% ethidium bromide-stained agarose gel. A threshold cycle value (*C*_T_) was estimated using the ∆∆*C*_T_ method to quantify the assessment of gene expression.

### Transfection of Akt small interfering RNA (siRNA)

The Akt siRNA and corresponding controls (Genepharma, generated) were mixed with TransMessenger Transfection Reagent (Qiagen, 301525) and administered to NMCMs according to protocol. After 4 h, the culturing media was replaced by normal culture media without antibiotics for 24 h. Then the cells were treated with poly(I:C) or vehicle for 12 h, after that, the cells were challenged with OGD performance (4 h of oxygen and glucose deprivation and 3 h of reoxygenation).

### Immunoprecipitation

The tissue samples and cells were washed twice with ice-cold PBS and lysed with cell lysis buffer (10 mM HEPES pH 7.9, 10 mM KCl, 1.5 mM MgCl_2_, 50 mM NaF 1 mM Na_3_VO_4_, 1 μM PMSF plus a protease inhibitor cocktail). Protein samples (800 μg) were separated on 10% SDS gel, transferred to polyvinylidene difluoride membranes, and incubated at 4 °C for 1 h with 2 μg antibodies to TLR3 (Abcam, ab62566) followed by the addition of 15 μl of protein G magnetic beads (Biorad, 161-4023). The precipitates were then washed four times with lysis buffer and subjected to immunoblotting (IB) with the antibody to p85 subunit PI3K (Cell Signaling Technology, 4257).

### Proximal ligation assay

PLA is an antibody-based technique to determine whether two proteins are with 40 nm of each other. Proteins detected in this manner are identifiable by fluorescence. Heart slides were fixed and incubated using Duolink^TM^ In Situ mouse/rabbit red starter kit (Sigma, DUO92101) according to protocols. Heart slides incubated with a blocking solution in a humidity chamber at 37 °C for 30 min. After removing the blocking solution, the primary antibody anti-TLR3 and anti-PI3K were added to cover each section and incubated at 4 °C overnight. Then, slides were washed twice with 5% bovine serum albumin and incubated with PLUS antibody and MINUS antibody at room temperature for 20 min. The secondary antibody mix was added and incubated at 37 °C for 1 h. The ligation mix was added and incubated at 37 °C for 30 min, then washed with 1× buffer A twice for 2 min. The amplification mix was added and incubated at 37 °C for 100 min. Slides were washed with 1× buffer A and B twice for 10 min and 0.01× buffer B for 1 min. Then slides were mounted in mounting medium with DAPI. Finally, slides were photographed with a DAPI-filter and 43 HE-filter to identify the cells and PLA reaction using a fluorescence microscope and images were analyzed with Image J software.

### NF-κB activation

NF-κB activation was quantified employing a p65 Transcription Factor Assay Kit (Abcam, ab133112). A nuclear extraction kit (Abcam, ab113474) was used first to extract the nuclear protein of H9C2. A 96-well plate was coated with a DNA binding sequence specific for the active form of NF-κB. Ten microlitre of nuclear extract were loaded into each designated well. After incubation and washing, the plate was incubated with antibody to p65 NF-κB. After another circle of incubation and washing, horseradish peroxidase conjugated secondary antibody was added to the wells, then developed for luminescence.

### Western blot

After the mice were sacrificed, the hearts were removed (*n* = 6/group). Total protein was isolated from ischemic myocardial tissue. The proteins were separated through a 12% sodium dodecyl sulfate-polyacrylamide gel electrophoresis and transferred to polyvinylidene difluoride membranes (Merck Millipore, ISEQ00010). The membrane was blocked and probed with primary antibodies overnight, including TLR3, PI3 kinase p85 (PI3K), phospho-PI3K (p-PI3K), Akt, phospho-Akt (p-Akt), p70 S6 kinase, Bax, Bcl-2, caspase3 and β-Actin at 1:500 dilutions, except for β-Actin (1:1000) (Supplementary Table 2). The membrane was then incubated with the secondary antibodies (ZSGB-Bio, ZB2301). The signals were scanned for the densitometry using a bioimaging analysis system (Bio-rad, ChemiDoc MP). The final blots were quantified with Image J 1.48v software, and the protein levels were standardized against loading control.

### Statistical analysis

Investigators were blinded to medication during analyses. Data are shown as mean ± SEM. The presented data were represented from at least six separate tests. Statistical analysis was performed with GraphPad Prism 8.0. Group mean values (two groups) comparison were carried out by two-unpaired Student’s *t* test. Multiple groups comparisons were performed using one-way ANOVA with the Tukey’s, Dunnett’s or Bonferroni’s multiple comparisons test (details in Figure legends). *P* values ≤ 0.05 are considered statistically significant.

## Supplementary information

Supplementary Materials

## Data Availability

The data that support the findings of this study are available from the authors on reasonable request, see author contributions for specific data sets.
