# Supplementary material for: Poly(I:C) preconditioning protects the heart against myocardial ischemia/reperfusion injury through TLR3/PI3K/Akt-dependent pathway
Source: Signal Transduct Target Ther. 2020 Nov 6;5:216. doi: 10.1038/s41392-020-00257-w (PMC7644758; doi:10.1038/s41392-020-00257-w)
Supplement: Supplementary file 1 — Supplementary Materials [file 41392_2020_257_MOESM1_ESM.docx]

Supplementary Materials for

Poly(I:C) preconditioning protects the heart against myocardial ischemia/reperfusion injury through TLR3/PI3K/Akt-dependent pathway

Erya Chen, Chan Chen, Zhendong Niu, Lu Gan, Qiao Wang, Ming Li, XingWei Cai, Rui Gao, Sruthi Katakam, Hai Chen, Shu Zhang, Ronghua Zhou, Xu Cheng, Yanhua Qiu, Hai Yu, Tao Zhu, Jin Liu

Correspondence to: [xychenchan@gmail.com](mailto:xychenchan@gmail.com)

**This PDF file includes:**

Figures. S1 to S5

Tables S1 to S2


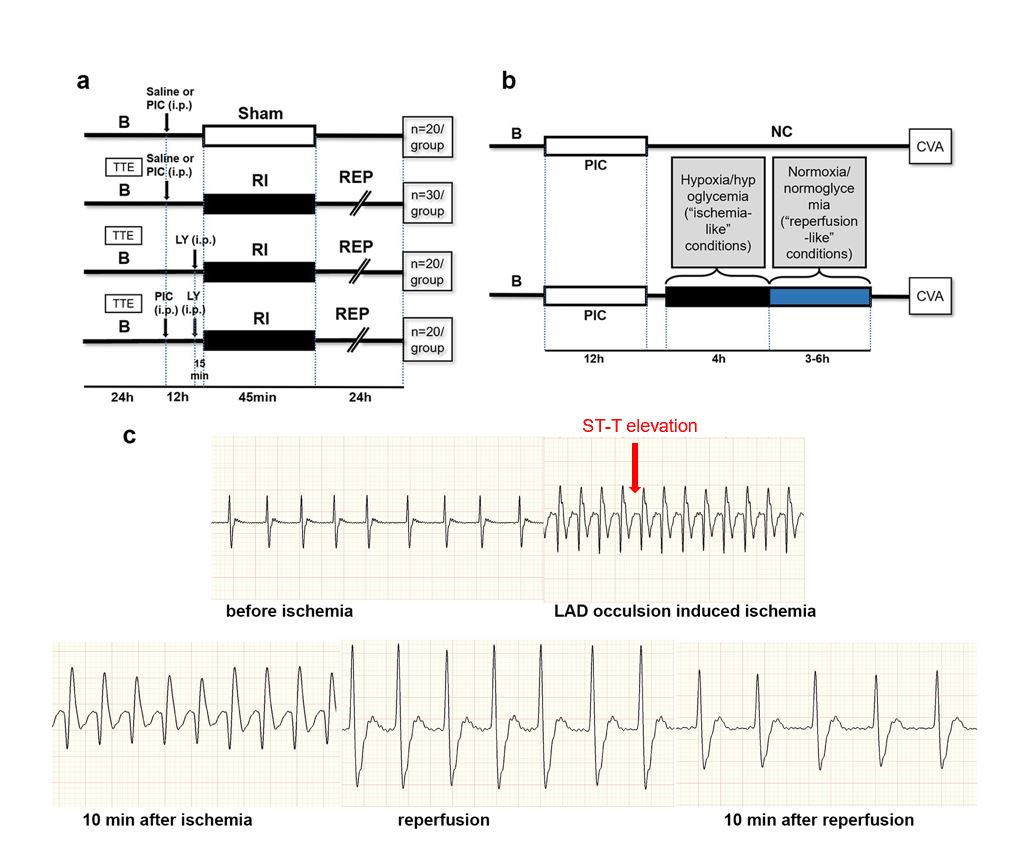


Figure. S1.

**Scheme of the experimental design and ECGs of ischemia/reperfusion procedure.**

(**a**) C57BL/6 mice underwent myocardial I/R with poly(I:C) and/or LY294002 preconditioning. (**b**) H9c2 cells were exposed to hypoxia/hypoglycemia conditions for 4 h and then suddenly exposed to normoxia/normoglycemia conditions for an additional 3-12 h. Abbreviations: B, baseline; RI, regional ischemia; REP, reperfusion; PIC, poly(I:C), LY, LY294002, TTE, transthoracic echocardiography; IS, infarct size; NC, normal culture; CVA, cell viability assay. (**c**)Representative ECG images before ischemia, during ischemia, upon reperfusion, and after reperfusion. Abbreviation: LAD, left anterior descending coronary artery.


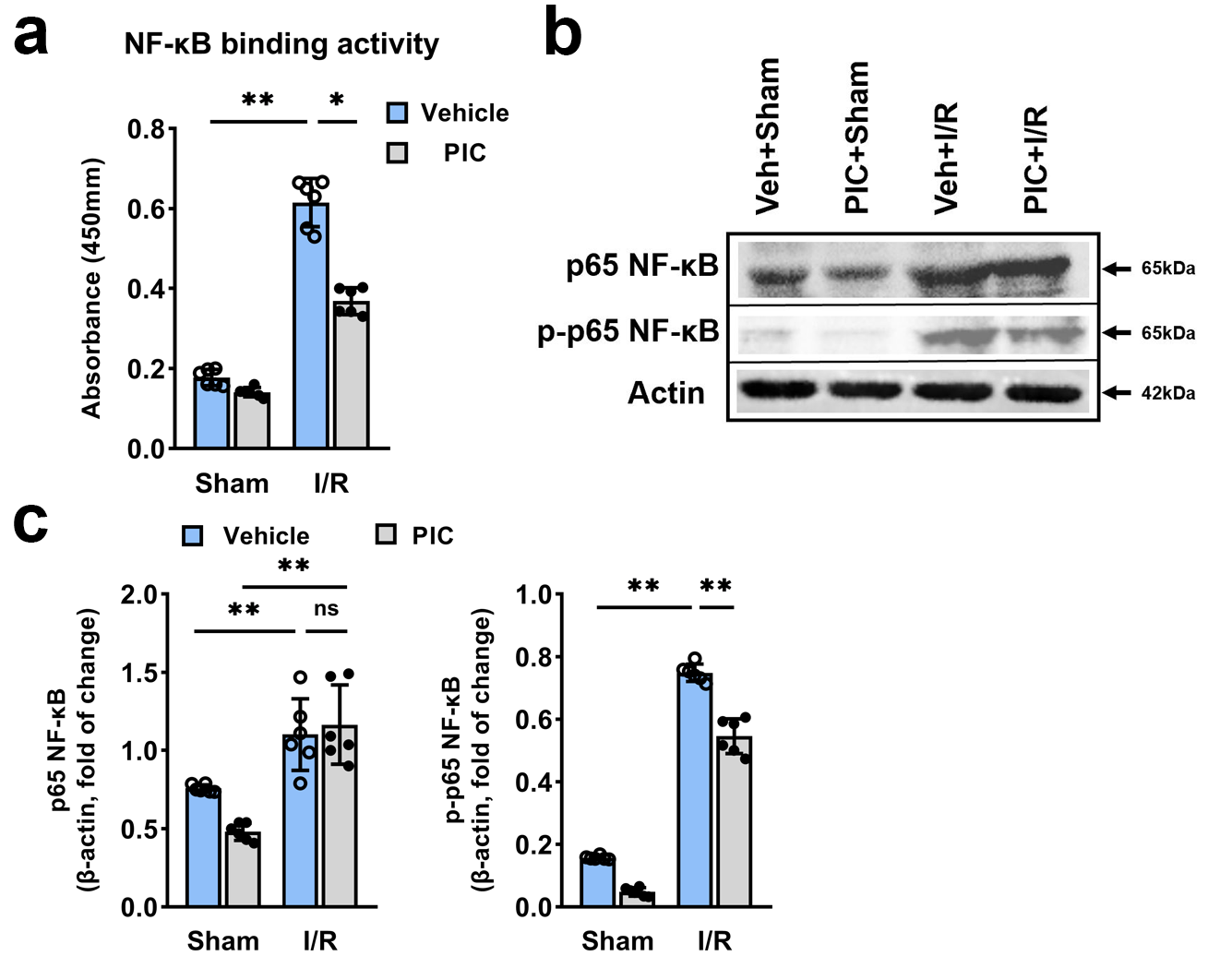


Figure. S2.

**The binding activity and protein expression level of NF-κB**

(**a**)The p65 NF-κB binding activity in ischemic myocardium in the presence or absence of poly(I:C) pre-treatment subjected to I/R (n=6). (**b-c**) Representative western blot (**b**) and average data (**c**)for phospho-p65 NF-κB, total p65 NF-κB in hearts subjected to sham and I/R with poly(I:C) or vehicle pre-treatment(n=6) (All experiment groups were compared via one-way ANOVA with a Bonferroni’s multiple comparisons test, bars indicate the SEM, **P* < 0.05; ***P* < 0.01). Abbreviations: ns, not statistically significant.


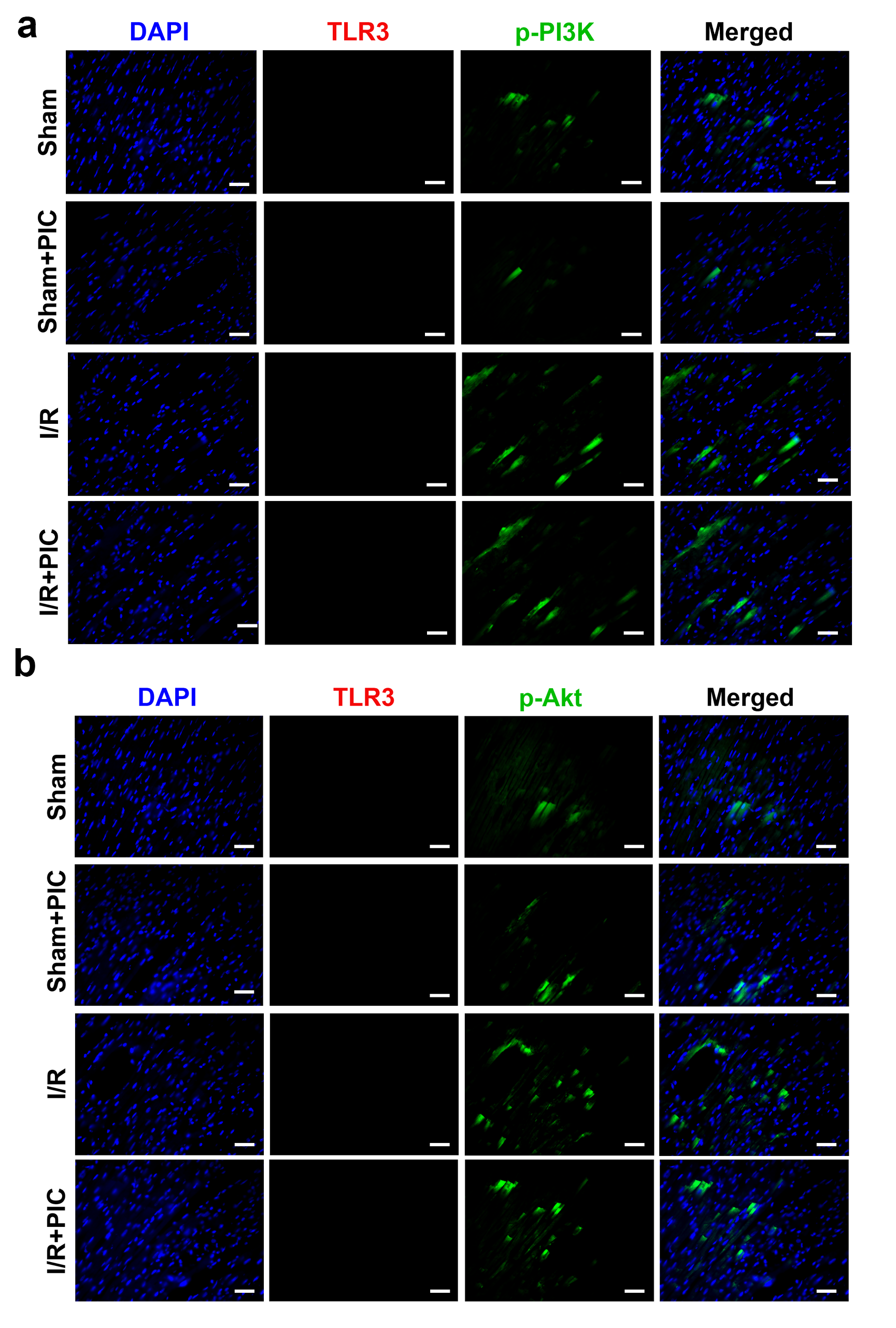


Figure. S3.

**Colocalization of TLR3 and phospho-PI3K or phospho-Akt in *tlr3^-/-^* mice ischemic myocardium.**

(**a**) Representative photographs of TLR3 and phospho-PI3K in *tlr3^-/-^* mice ischemic myocardium by immunofluorescence colocalization. Scale bars, 50μm. (**b**) Representative photographs of TLR3 and phospho-Akt in *tlr3^-/-^* mice ischemic myocardium by immunofluorescence colocalization. Scale bars, 50μm.


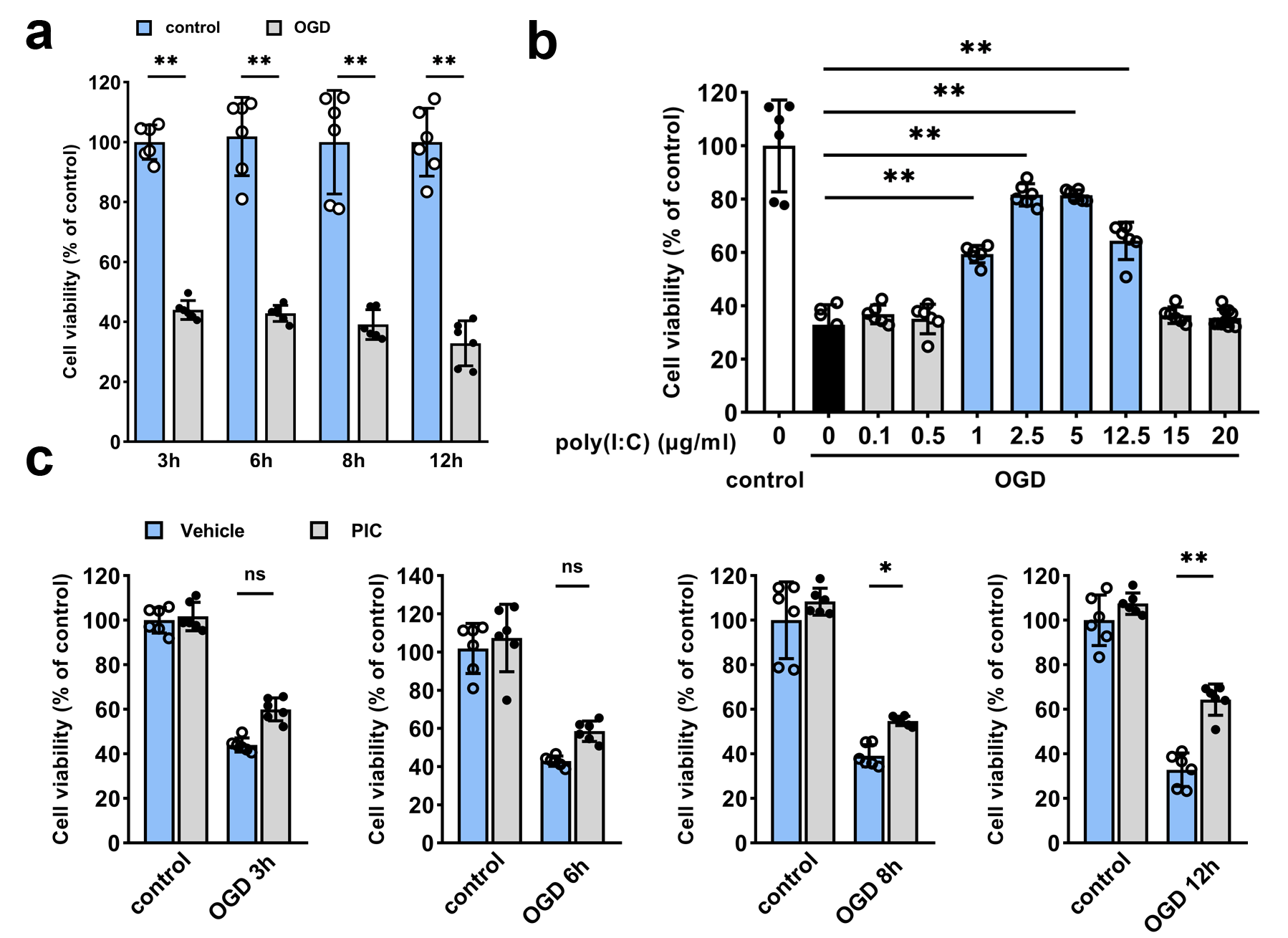


Figure. S4.

**The OGD time period and different concentrations of poly(I:C) in H9c2 cell lines.**

(**a**) Cells viability decreased during the different periods of OGD (n=6) (All experiment groups were compared between control and OGD groups via unpaired Student’s *t* test, bars indicate the SEM, **P* < 0.05; ***P* < 0.01). (**b**) Cell viability with poly(I:C) pretreatment (0.1 to 100 μg/ml) 12 h before 4 h OGD following 12 h reoxygen (n=6)(All experiment groups were compared with 0 μg/ml poly(I:C) group via unpaired Student’s *t* test, bars indicate the SEM, **P* < 0.05; ***P* < 0.01). (**c**) The different periods of reoxygen (3 h, 6 h, 8 h, 12 h) after 4 h OGD with poly(I:C) 12.5 μg/ml and change of the cell viability (n=6) (All experiment groups were compared via one-way ANOVA with a Bonferroni’s multiple comparisons test, bars indicate the SEM, **P* < 0.05; ***P* < 0.01).


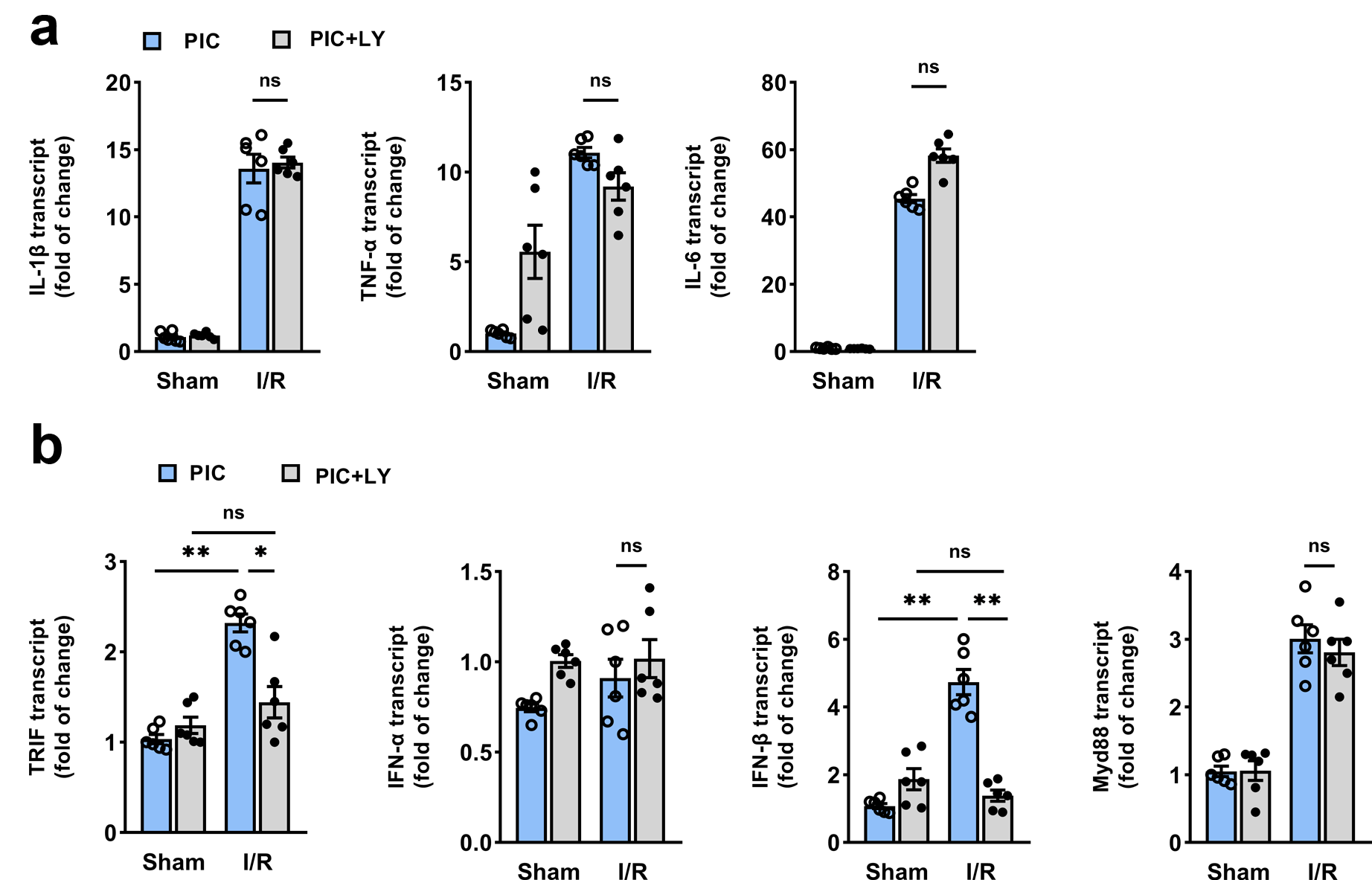


Figure. S5.

**Inflammatory factors and LR3 downstream molecule gene expression after poly(I:C) and LY294002 preconditioning**

(**a**) Expression of inflammatory cytokine IL-1β, TNF-α, and IL-6 as assessed qRT-PCR in hearts subjected to sham and I/R with poly(I:C) and/or LY294002 pre-treatment (n=6). (**b**) Expression of TRIF, IFN-α, IFN-β, and Myd88 as assessed by qRT-PCR in hearts subjected to sham and I/R with poly(I:C) and/or LY294002 pre-treatment (n=6). (All experiment groups were compared via one-way ANOVA with a Bonferroni’s multiple comparisons test, bars indicate the SEM, **P* < 0.05; ***P* < 0.01).

Table S1.

**Primers used in the qRT-PCR assay.**

| **Name** | **5’-3’sequence** | | |
| --- | --- | --- | --- |
|  | Forward primer | Reverse primer | |
| tnf-α | CTGTGAAGGGAATGGGTGTT | | CAGGGAAGAATCTGGAAAGGTC |
| il-1β | TGCCACCTTTTGACAGTGATG | | CAGGGAAGAATCTGGAAAGGTC |
| il-6 | TGAGAAAAGAGTTGTGCAATGG | | GGAGAGCATTGGAAATTGGGG |
| ifn-α | GACCTGCAAGGCTGTCTGAT | | AGACAGGGCTCTCCAGACTT |
| tlr3 | CCTGCTGGAAAACTGGATGGC | | CCCTTTCATGATTCAGCCCAGA |
| trif | CCTGAGCCTGCATCAAATC | | CCACCTTTCTGGCGAAGA |
| ifn-β | CCACCTTTCTGGCGAAGA | | TCTCTGCTCGGACCACCAT |
| tlr4 | ACCTGGCTGGTTTACACGTC | | GTGCCAGAGACATTGCAGAA |
| myd88 | ACTCGCAGTTTGTTGGATG | | CACCTGTAAAGGCTTCTCG |

Table S2.

**Details of antibodies used in this study.**

| **Name** | | **Company** | **Item No.** |
| --- | --- | --- | --- |
| TLR3 | Abcam | | ab62566 |
| PI3K | Cell Signaling Technology | | #4257 |
| p-PI3K | Cell Signaling Technology | | #4228 |
| Akt | Cell Signaling Technology | | #4691 |
| p-Akt | Cell Signaling Technology | | #4060 |
| p70 S6 kinase | Cell Signaling Technology | | #9202 |
| BAX | Cell Signaling Technology | | #5023 |
| Bcl-2 | Proteintech | | 12789-1-AP |
| Caspase 3 | Proteintech | | 66470-2-lg |
| PY20 | Abcam | | ab10321 |
| NF-κB | Abcam | | ab16502 |
| p-NF-κB | ZBS | | 310013 |
| TRIF | Abcam | | ab13810 |
| TLR4 | Abcam | | ab13556 |
| β-Actin | Proteintech | | 60008-1-lg |
